# The proportion, clinical predictors, and prognostic impact of hypometabolic estrogen receptor–positive primary breast cancer on baseline [^18^F] fluorodeoxyglucose PET

**DOI:** 10.1097/MNM.0000000000002133

**Published:** 2026-02-25

**Authors:** Melissa Lenaerts, Lois van der Voort, Marjolein L. Smidt, Tineke van de Weijer, Felix M. Mottaghy, Adri C. Voogd, Sandra M.E. Geurts, Vivianne C.G. Tjan-Heijnen, Ferdia A. Gallagher, Luigi Aloj, Thiemo J.A. van Nijnatten

**Affiliations:** a Department of Surgery, Maastricht University Medical Center; b GROW – Research Institute for Oncology and Reproduction, Maastricht University; c Department of Radiology and Nuclear Medicine, Maastricht University Medical Center+, Maastricht, The Netherlands; d Department of Nuclear Medicine, University Hospital RWTH Aachen University, Aachen, Germany; e Department of Epidemiology, Maastricht University; f Department of Medical Oncology, Maastricht University Medical Center+, Maastricht, The Netherlands; g Department of Radiology, School of Clinical Medicine, University of Cambridge, Cambridge, UK

**Keywords:** breast cancer, estrogen receptor, [^18^F] fluorodeoxyglucose PET, hypometabolic, staging

## Abstract

**Objective:**

Previous studies reported low [^18^F] fluorodeoxyglucose ([^18^F]FDG) PET uptake in estrogen receptor–positive breast tumours, potentially missing detection of distant metastases. This study assessed the proportion of estrogen receptor–positive hypometabolic tumours, clinical factors influencing [^18^F]FDG uptake, and the prognostic impact.

**Methods:**

Baseline [^18^F]FDG PET/computed tomography (CT) and [^18^F]FDG PET/MRI exams of female patients diagnosed with estrogen receptor–positive locally advanced (cT3-4N0 or cT1-4N+), metastatic, or recurrent breast cancer between 2013–2022 were retrospectively collected. Different thresholds of maximum standardised uptake value (SUV_max_) and tumour-to-background ratio (TBR; SUV_max_ tumour/SUV_max_ background) were applied to determine the proportion of hypometabolic [^18^F]FDG PET exams. Logistic regression and survival analysis were performed.

**Results:**

119 patients underwent [^18^F]FDG PET/CT and 31 [^18^F]FDG PET/MRI. The proportion of hypometabolic tumours for SUV_max_ thresholds 2.0, 2.5, 3.0, TBR of contralateral breast less than or equal to 1, and TBR of liver less than or equal to 1 was 8.4, 15.1, 21.8, 5.1, and 28.6%, respectively for [^18^F]FDG PET/CT and 16.1, 19.4, 29.0, 6.9, and 35.5% for [^18^F]FDG PET/MRI. Clinically tumour status (cT-status), histology type, and tumour grade were associated with the presence of a hypometabolic tumour. No PET-derived variables were associated with recurrence-free survival.

**Conclusion:**

A considerable proportion of estrogen receptor–positive breast tumours showed low SUV_max_, indicating potential suboptimal staging on [^18^F]FDG PET. In patients with lower cT-status, lobular histology and low-grade estrogen receptor–positive tumour, [^18^F]FDG PET may be less reliable as staging procedure. Further research is necessary to determine the optimal metabolic threshold for defining a hypometabolic tumour.

## Introduction

Breast cancer is the most commonly diagnosed cancer in women globally, including approximately 2.3 million new cases worldwide in 2022 [1,2]. The prognosis of these patients varies according to the extent of breast cancer. Ten-year breast cancer-specific survival rates decline substantially from 96% in patients with stage I breast cancer to 13% in stage IV breast cancer [3].

Adequate staging in patients presenting with stage IIB through stage IV breast cancer is essential for guiding therapeutic decision-making. Current European Association of Nuclear Medicine (EANM) and Society of Nuclear Medicine and Molecular Imaging guidelines recommend the use of [^18^F] fluorodeoxyglucose ([^18^F]FDG) PET for distant staging in patients with locally advanced breast cancer (LABC) [4]. [^18^F]FDG PET may be combined with computed tomography (CT) or MRI to obtain morphologic information [5–7]. On PET, the [^18^F]FDG uptake, reflecting the metabolic activity of the tumour, is typically assessed by measuring the maximum standardised uptake value (SUV_max_) [8].

Previous studies found differences in [^18^F]FDG uptake between breast cancer clinical subtypes [9,10]. A recent meta-analysis by de Mooij *et al*. [10] reported a significantly lower metabolism in the primary breast tumour on [^18^F]FDG PET/CT in patients with estrogen receptor–positive breast cancer compared to those with estrogen receptor–negative breast cancer. Estrogen receptor–positive breast cancer represents approximately 70–80% of invasive breast cancer [11–13]. Within histological subtypes, invasive carcinoma of no special type (NST) is estrogen receptor–positive in approximately 70% of cases, whereas more than 90% of invasive lobular carcinoma (ILC) express estrogen receptor–positivity [14–16]. Other studies have reported that smaller tumour size, lower tumour grade, absence of p53 mutations, and ILC are associated with lower [^18^F]FDG uptake [17,18]. A primary tumour with a low [^18^F]FDG uptake (i.e. a hypometabolic tumour) may potentially be accompanied by hypometabolic metastases, which can be difficult to detect on [^18^F]FDG PET imaging. Failure to detect metastases can result in understaging of the disease, as was demonstrated in a study by Iqbal *et al*. [9], in which 7.1% (19/267) of grade 1–2 estrogen receptor–positive lesions [breast tumour, axillary lymph nodes (ALNs), and distant metastases] were classified as false negative during staging with [^18^F]FDG PET/CT. Eventually prognosis of patients with decreased detection of metastases on [^18^F]FDG PET may be affected because of relatively late treatment in these cases, rather than early detection with appropriate treatment possibilities.

In the literature, the definition of a hypometabolic tumour remains unclear, as most studies only investigated the SUV_max_ threshold for benign vs. malignant breast lesions [19]. No standardised threshold for low metabolic activity has been established yet. Therefore, in this study, multiple metabolic thresholds were examined. For every metabolic threshold, this study aimed to investigate the proportion of women diagnosed with estrogen receptor–positive breast cancer who have a hypometabolic uptake on [^18^F]FDG PET in the primary tumour. Furthermore, we assessed clinical factors that influence [^18^F]FDG uptake and the prognosis of hypometabolic tumours at varying metabolic thresholds.

## Methods

### Study design

In this single-centre retrospective study, female patients diagnosed between 2013 and 2022 with biopsy-proven estrogen receptor–positive (estrogen receptor percentage ≥ 10%) LABC, metastatic, or recurrent breast cancer were included. LABC was defined as invasive breast cancer with a tumour larger than 5 cm and ALN metastasis (cT3-4N0 or cT1-4N+) [20]. Patients were eligible if they underwent an [^18^F]FDG PET/CT or [^18^F]FDG PET/MRI exam at the time of breast cancer diagnosis. In case of bilateral estrogen receptor–positive breast cancer, both sides were included separately. Male patients were excluded, as well as patients who had a diagnosis of inflammatory breast cancer, occult breast cancer (*n* = 4), or if their baseline [^18^F]FDG PET scan was of insufficient quality to permit the PET analyses (*n* = 2). This study was conducted in accordance with the principles of the Declaration of Helsinki and was approved by the local medical ethics research committee (METC 2022-3480), which waived the requirement for informed consent.

### [^18^F] fluorodeoxyglucose PET imaging

Patients underwent an [^18^F]FDG PET/CT (Gemini TF; Philips Healthcare, Best, The Netherlands until and including 2018/MI5 Discovery; GE Healthcare, Eindhoven, The Netherlands from 2019 onwards) or [^18^F]FDG PET/MRI (Biograph mMR; Siemens Healthcare, Erlangen, Germany; since 2015) exam in Maastricht UMC+ (Maastricht, The Netherlands) according to the standard acquisition protocol [21,22]. Patients were required to fast for at least 4 h before [^18^F]FDG administration and remain well-hydrated. If the serum glucose concentration was below 11 mmol/l (in one patient, PET imaging was performed despite a serum glucose level of 12 mmol/l after discussion with the nuclear medicine physician), [^18^F]FDG was intravenously administered with a dose of 2–3 MBq/kg of body weight. The [^18^F]FDG PET/CT or [^18^F]FDG PET/MRI scan was carried out 45–60 min after [^18^F]FDG administration. Intensive exercise was prohibited from 24 h before [^18^F]FDG administration until completion of the PET exam. For [^18^F]FDG PET/CT, patients were placed in the scanner in a supine position with elevated arms, allowing for a standard supine whole-body [^18^F]FDG PET/CT scan (field of view extended from skull vertex to pelvis). For both the Philips Healthcare (2013–2018) and GE Healthcare (2019–2022) PET/CT scanners, images were reconstructed according to EANM Research Ltd. (EARL) 1 guidelines [4,21,22]. For [^18^F]FDG PET/CT 2013–2018, the BLOB-OS time-of-flight algorithm was used to reconstruct the PET images, using a voxel size of 4 × 4 × 4 mm^3^. CT images were reconstructed using filtered-back projection (tube voltage of 120 kV, effective tube current of 30 mAs). For [^18^F]FDG PET/CT 2019–2022, ordered-subsets expectation-maximization reconstruction with two iterations, 17 subsets, and a Gaussian postsmoothing filter of 7 mm was used. CT images were made with a tube voltage of 140 kV, dose modulation with a noise index of 50%, and an effective tube current of 10–60 mA.

For [^18^F]FDG PET/MRI, patients were placed in a prone position with both arms above their head (field-of-view extended from skull base to pelvis). All PET images were reconstructed according to EARL 1 guidelines using ordered-subsets expectation-maximization with three iterations, 21 subsets, and a Gaussian postsmoothing of 5 mm. The MRI protocol consisted of a two-dimensional T2-weighted turbo spin-echo sequence without fat suppression, a diffusion-weighted imaging sequence with fat suppression, and a dynamic contrast-enhanced T1-weighted sequence with fat suppression. Gadobutrol (Gadovist; Bayer Health Care, Leverkusen, Germany) was used as the MRI contrast agent, injected through a catheter in the antecubital vein at a 0.1 mmol/kg bodyweight, followed by a saline flush [23]. The treatment regimen can be found in Supplementary File S1, Supplemental digital content 1, https://links.lww.com/NMC/A397.

### Imaging assessment

PET analyses were performed by a nuclear medicine physician (T.J.A.v.N.) with 4 years of clinical experience in [^18^F]FDG PET reporting. A lesion was characterised as malignant if it visually showed a focally increased [^18^F]FDG uptake compared with the surrounding tissue. The SUV_max_ and SUV_mean_ of the primary tumour, most hypermetabolic ALN and background (contralateral breast and liver parenchyma), and the number of hypermetabolic ALNs were prospectively evaluated using Sectra (Sectra AB, Linköping, Sweden). Analysis was performed separately for [^18^F]FDG PET/CT and [^18^F]FDG PET/MRI to account for technical differences between the two imaging modalities.

### Data collection

Data were retrospectively extracted from the electronic patient record. Variables collected included age at diagnosis, primary tumour size, clinical TNM status, breast cancer stage (American Joint Committee on Cancer), multifocality, histology type, estrogen receptor percentage, progesterone receptor status, human epidermal growth factor receptor 2 (HER2) status, tumour grade [modified Bloom–Richardson grade (mBR)], presence of lymphovascular invasion, treatment type [primary surgery vs. neoadjuvant chemotherapy (NAC)], breast and axillary surgery type and details, and pathological response following NAC. In some cases, HER2 status and tumour grade (mBR) were not determined on the pathological biopsy and were therefore recorded as missing. These patients were retained in the study as their inclusion did not affect the primary objective of the analysis. For survival analyses, follow-up data were retrospectively extracted from the electronic patient record up to 7 February 2025.

### Statistical analysis

Statistical analyses were performed using Statistical Package for the Social Sciences (SPSS; Version 28; IBM Corp, Armonk, New York, USA). Graphs were made using GraphPad Prism (v5.1; GraphPad Software, San Diego, California, USA). Continuous characteristics are described as mean ± SD or median (range) and compared using the unpaired *t*-test. Categorical variables are described as absolute numbers and percentages, and compared using the *χ*^2^ test. The proportion of patients with hypometabolic [^18^F]FDG uptake was determined based on multiple metabolic thresholds. Three SUV_max_ thresholds (2.0, 2.5, and 3.0) were applied, followed by the tumour-to-background ratio (TBR) of the contralateral breast and liver parenchyma (SUV_max_ of the primary tumour divided by the SUV_max_ of the contralateral breast or liver parenchyma; threshold ≤ 1). Univariable and multivariable logistic regression were performed for all metabolic thresholds to determine factors that influence the metabolic activity of the primary tumour. Associations are expressed as odds ratios (OR) with 95% confidence interval (CI) and *P* values. Parameters with a significant association were entered simultaneously in multivariable logistic regression to evaluate the independent value of each parameter after adjustment for the other parameters. Multivariable logistic regression was performed when at least 10 patients fell in the category below the threshold. The maximum number of variables in the multivariable model was based on the assumption that one variable per five events can be added to the model [24]. Recurrence-free survival (RFS) was analysed using a Kaplan–Meier analysis. RFS was defined as the time from diagnosis until local, regional, or contralateral recurrence, distant metastasis, breast cancer-related mortality, or last follow-up, with recurrence determined based on clinical, imaging, or biochemical evidence, whichever occurred first. The log-rank test was used to compare RFS between groups. A Cox proportional hazards model was used to determine variables associated with RFS. The prognostic value of the covariates in the model was quantified by the hazard ratio with 95% CI and *P* values. The Cox proportional hazards model was performed only when at least 10 events occurred during follow-up [25,26]. The proportional hazard assumption was tested using R Statistical Software (v4.3.1; R Core Team 2023, Vienna, Austria). Analyses of [^18^F]FDG PET/CT and [^18^F]FDG PET/MRI were conducted separately to account for technical differences between the two imaging modalities. *P* values less than 0.05 (two-tailed) were considered statistically significant.

## Results

### Patient characteristics

**Fig. 1:**
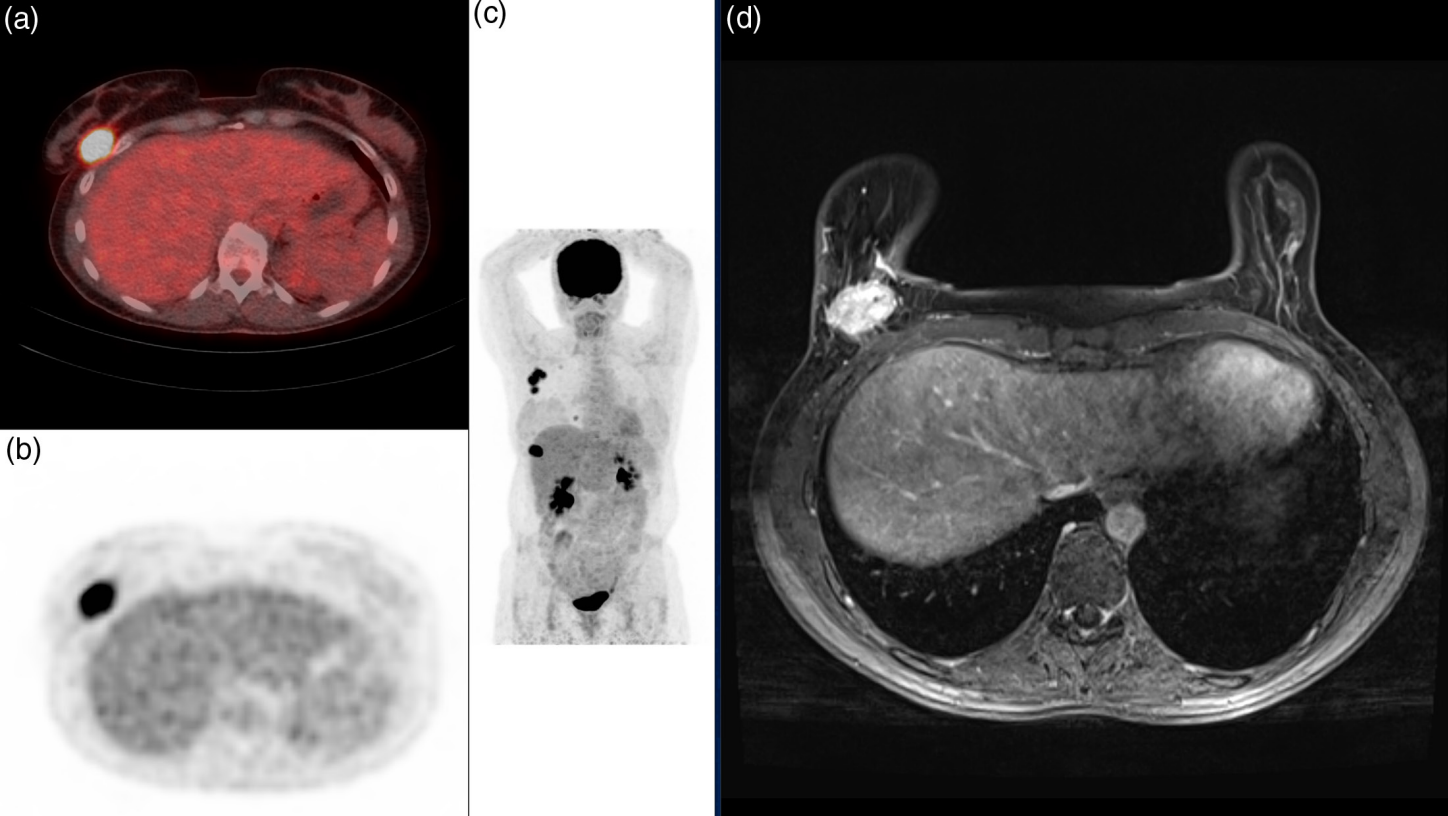
Baseline [^18^F]FDG PET/CT; scan of a 50-year-old patient with right estrogen receptor–positive breast cancer of no special type with a high metabolic uptake (SUV_max_ primary tumour = 9.25). The breast lesion was detected on [^18^F]FDG PET/CT (a, axial fusion image; b, axial PET image; c, maximum intensity projection image) and on MRI (d, axial contrast-enhanced T1-weighted MRI exam). CT, computed tomography; [^18^F]FDG, [^18^F] fluorodeoxyglucose; SUV_max_, maximum standardised uptake value.

**Fig. 2:**
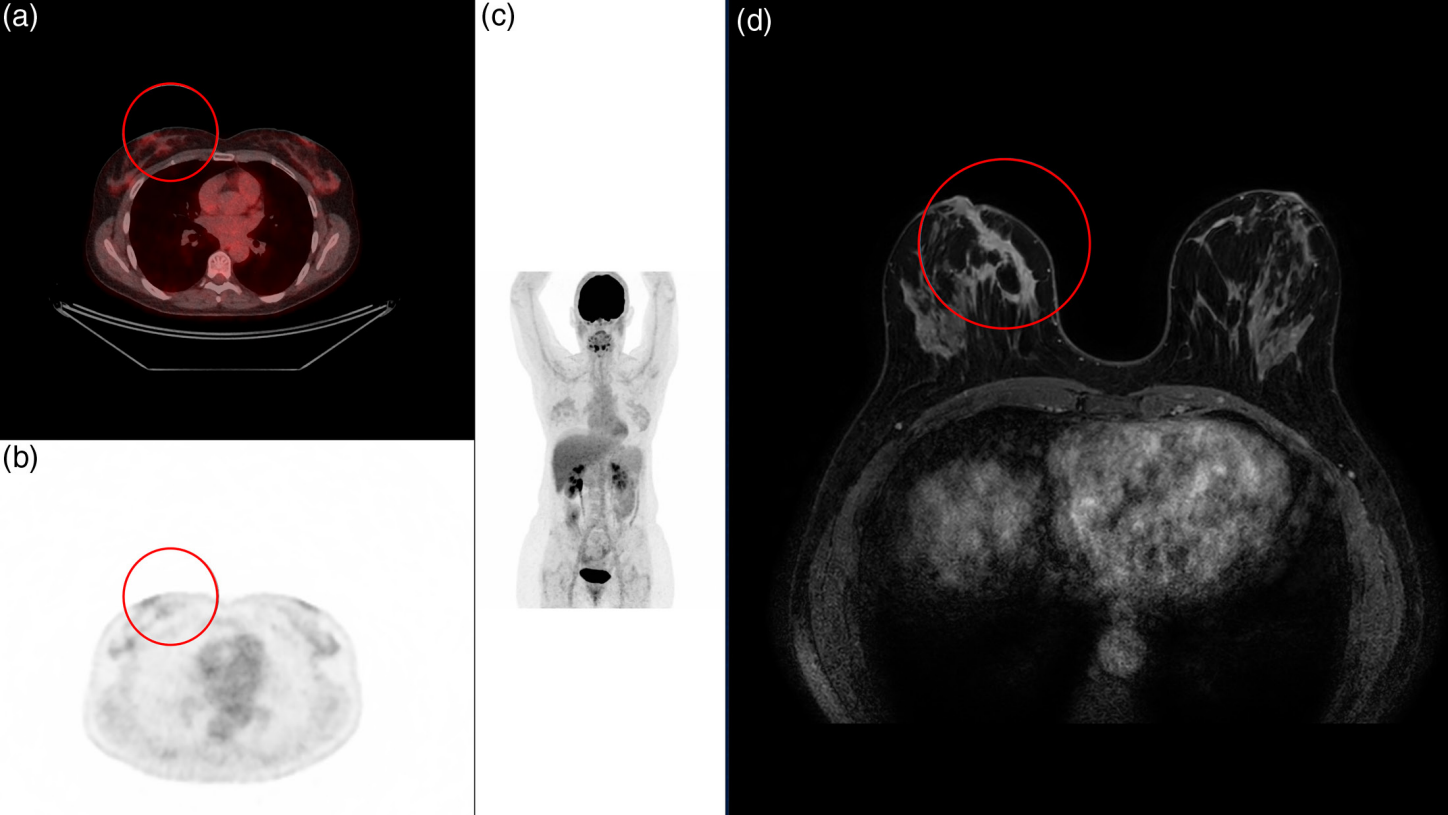
Baseline [^18^F]FDG PET/CT exam of a 39-year-old patient with right estrogen receptor–positive lobular breast cancer with a low metabolic uptake (SUV_max_ primary tumour = 1.53). The breast lesion was not visible on [^18^F]FDG PET/CT (a, axial fusion image; b, axial PET image; c, maximum intensity projection image), but was detected on MRI (d, axial contrast-enhanced T1-weighted MRI exam). CT, computed tomography; [^18^F]FDG, [^18^F] fluorodeoxyglucose; SUV_max_, maximum standardised uptake value.

In total, 150 female patients diagnosed with estrogen receptor–positive LABC, metastatic, or recurrent breast cancer between 2013 and 2022 were included in this study, of whom 119 underwent an [^18^F]FDG PET/CT exam and 31 an [^18^F]FDG PET/MRI exam (Figs. 1 and 2). Patient and tumour demographics at baseline are shown in Table 1. The mean age was 57 years for both the [^18^F]FDG PET/CT and [^18^F]FDG PET/MRI group. Of all patients, 70.7% (106/150) had HER2-negative and 26.7% (40/150) had HER2-positive breast cancer. A total of 15.3% (23/150) of patients underwent primary surgery; all other patients were treated with NAC (84.7%, 127/150). In Table 2, metrics of PET-related characteristics are provided for [^18^F]FDG PET/CT and [^18^F]FDG PET/MRI separately.

**Table 1:** Patient and tumour demographics

Characteristic	Total (*n* = 150)	[^18^F]FDG PET/CT (*n* = 119)	[^18^F]FDG PET/MRI (*n* = 31)	*P* value
Age at diagnosis (mean ± SD)	56.6 ± 12.6	56.5 ± 12.4	56.7 ± 13.3	0.934
Primary tumour size	40.7 ± 21.7	41.8 ± 22.4	36.6 ± 18.8	0.232
cT-status, *n* (%)				0.530
cT1	21 (14.0)	16 (13.4)	5 (16.1)	
cT2	68 (45.3)	51 (42.9)	17 (54.8)	
cT3	40 (26.7)	34 (28.6)	6 (19.4)	
cT4	21 (14.0)	18 (15.1)	3 (9.7)	
cN-status, *n* (%)				0.649
cN0	21 (14.0)	16 (13.4)	5 (16.1)	
cN1	110 (73.3)	86 (72.3)	24 (77.4)	
cN2	3 (2.0)	3 (2.5)	0 (0.0)	
cN3	16 (10.7)	14 (11.8)	2 (6.5)	
cM-status, *n* (%)				0.325
cM0	139 (92.7)	109 (91.6)	30 (96.8)	
cM1	11 (7.3)	10 (8.4)	1 (3.2)	
Breast cancer stage (AJCC)				0.828
Stage I	2 (1.3)	1 (0.8)	1 (3.2)	
Stage II	81 (54.0)	62 (52.1)	19 (61.3)	
Stage III	56 (37.3)	46 (38.7)	10 (32.3)	
Stage IV	11 (7.3)	10 (8.4)	1 (3.2)	
Multifocal tumour, *n* (%)	63 (42.0)	50 (42.0)	13 (41.9)	0.993
Histology type, *n* (%)				**0.039**
Invasive NST	126 (84.0)	100 (84.0)	26 (83.9)	
ILC	20 (13.3)	18 (15.1)	2 (6.5)	
Mixed invasive NST + ILC	1 (0.7)	0 (0.0)	1 (3.2)	
Other^a^	3 (2.0)	1 (0.8)	2 (6.4)	
ER% biopsy, median (range)	99 (10–100)	99 (10–100)	100 (60–100)	0.350
PR status, *n* (%)				0.512
Negative	51 (34.0)	42 (35.3)	9 (29.0)	
Positive	99 (66.0)	77 (64.7)	22 (71.0)	
HER2 status, *n* (%)				0.720
Negative	106 (70.7)	85 (71.4)	21 (67.7)	
Positive	40 (26.7)	31 (26.1)	9 (29.0)	
Missing	4 (2.7)	3 (2.5)	1 (3.2)	
Tumour grade (mBR), *n* (%)				0.836
Grade 1	16 (10.7)	13 (10.9)	3 (9.7)	
Grade 2	80 (53.3)	62 (52.1)	18 (58.1)	
Grade 3	49 (32.7)	40 (33.6)	9 (29.0)	
Missing	5 (3.3)	4 (3.4)	1 (3.2)	
Lymphovascular invasion, *n* (%)	14 (9.3)	12 (10.1)	2 (6.4)	0.316
Therapy, *n* (%)				0.326
NAC	127 (84.7)	99 (83.2)	28 (90.3)	
Primary surgery	23 (15.3)	20 (16.8)	3 (9.7)	
Surgery type				
* *Breast, *n* (%)				0.543
Lumpectomy	37 (24.7)	28 (23.7)	9 (29.0)	
Mastectomy	112 (74.7)	90 (76.3)	22 (71.0)	
* *Axilla, *n* (%)				**<0.001**
SLNB	25 (16.7)	21 (17.6)	4 (12.9)	
SLNB + MARI	31 (20.7)	14 (11.8)	17 (54.8)	
ALND	90 (60.0)	81 (68.1)	9 (29.0)	
None	4 (2.7)	3 (2.5)	1 (3.2)	
pCR after NAC, *n* (%)	28 (22.0)	23 (23.2)	5 (17.9)	0.545

Bold values indicate statistical significance (p < 0.05).

a Other: invasive carcinoma with apocrine features, mucinous carcinoma, and invasive carcinoma with apocrine/oncocytic features.

AJCC, American Joint Committee on Cancer; ALND, axillary lymph node dissection; cM-status, clinically metastatic status; cN-status, clinically nodal status; CT, computed tomography; cT-status, clinically tumour status; ER, estrogen receptor; [^18^F]FDG, [^18^F] fluorodeoxyglucose; HER2, human epidermal growth factor receptor 2; ILC, invasive lobular carcinoma; MARI, marking axillary lymph node with radioactive iodine seed; mBR, modified Bloom–Richardson grade; NAC, neoadjuvant chemotherapy; NST, no special type; pCR, pathologic complete response; PR, progesterone receptor; SLNB, sentinel lymph node biopsy.

**Table 2:** PET characteristics of [^18^F] fluorodeoxyglucose PET/computed tomography and [^18^F] fluorodeoxyglucose PET/MRI

Characteristic	*n*	[^18^F]FDG PET/CT Median (range)	*n*	[^18^F]FDG PET/MRI Median (range)
[^18^F]FDG dose (MBq)	118	155.8 (94–354)	30	205.5 (104–316)
Serum glucose (mmol/l)	117	5.4 (4.2–10.7)	31	5.2 (4.3–12.1)
Primary tumour	119		31	
SUV_max_		5.5 (1.1–27.7)		5.5 (0.5–18.9)
SUV_mean_		2.5 (0.5–15.5)		3.4 (0.3–9.7)
Most hypermetabolic ALN	118		31	
SUV_max_		3.8 (0.7–31.2)		2.0 (0.4–14.8)
SUV_mean_		1.6 (0.5–21.6)		1.4 (0.3–7.3)
Contralateral breast	118		29	
SUV_max_		1.6 (0.4–2.5)		1.1 (0.4–2.6)
SUV_mean_		0.6 (0.2–1.6)		0.6 (0.1–1.6)
Liver parenchyma	119		31	
SUV_max_		3.4 (2.1–6.0)		3.5 (2.7–6.4)
SUV_mean_		2.4 (1.2–3.9)		2.3 (1.6–3.0)

ALN, axillary lymph node; [^18^F]FDG, [^18^F] fluorodeoxyglucose; MBq, megabecquerel; SUV, standardised uptake value.

### Proportion of hypometabolic [^18^F] fluorodeoxyglucose uptake

#### Thresholds based on maximum standardised uptake value 2.0, 2.5, and 3.0

**Fig. 3:**
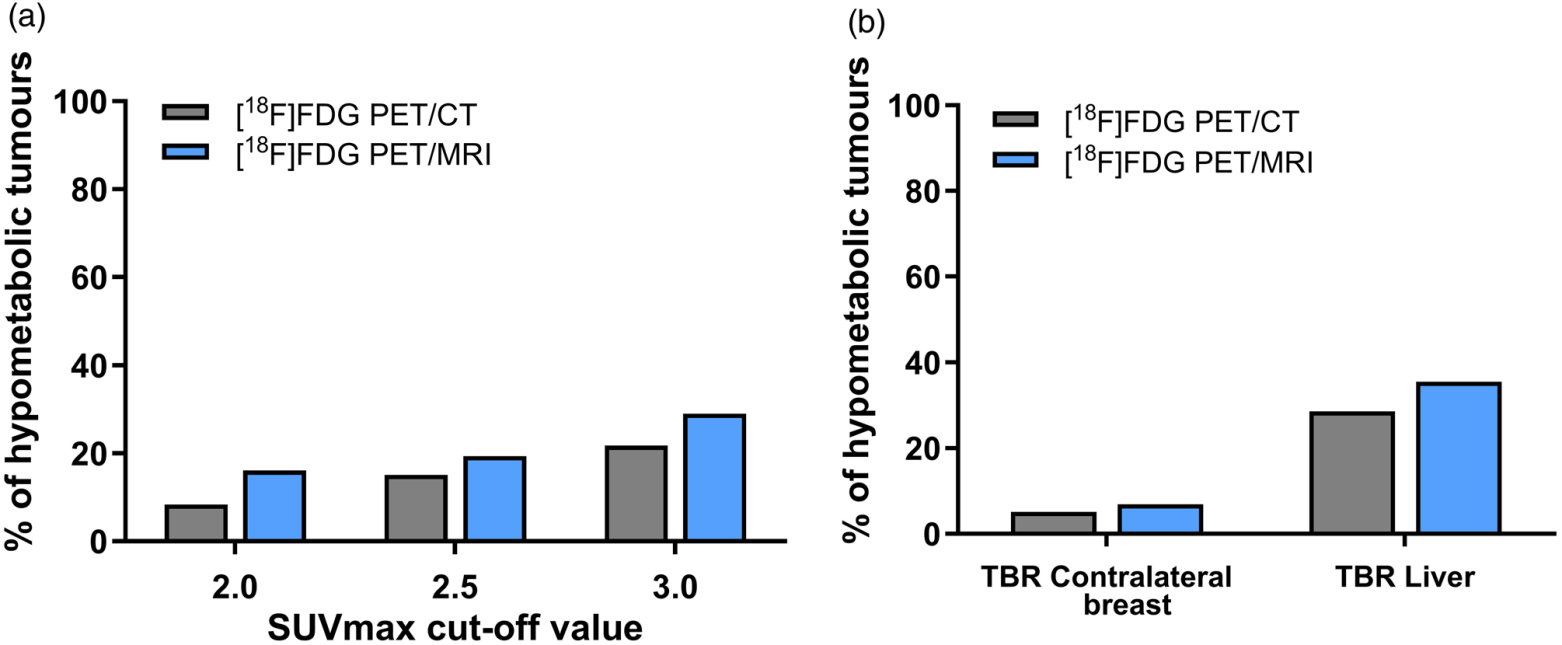
Percentage of patients with a SUV_max_ of the primary tumour below or equal to (a) SUV_max_ threshold 2.0, 2.5, and 3.0, and (b) with a TBR of the contralateral breast and liver parenchyma below or equal to 1 for [^18^F]FDG PET/CT (grey), and [^18^F]FDG PET/MRI (blue). CT, computed tomography; [^18^F]FDG, [^18^F] fluorodeoxyglucose; SUV_max_, maximum standardised uptake value; TBR, tumour-to-background ratio.

SUV_max_ thresholds 2.0, 2.5, and 3.0 corresponded with a proportion of women with a primary tumour SUV_max_ below or equal to the threshold of 8.4% (10/119), 15.1% (18/119), and 21.8% (26/119) for [^18^F]FDG PET/CT and 16.1% (5/31), 19.4% (6/31), and 29.0% (9/31) for [^18^F]FDG PET/MRI (Fig. 3a).

#### Threshold based on the tumour-to-background ratio of the contralateral breast

For [^18^F]FDG PET/CT, the mean TBR of the contralateral breast was 5.5, ranging from 0.8 to 37.1. In 5.1% (6/118), the SUV_max_ of the primary tumour was lower than or equal to the SUV_max_ of the contralateral breast (TBR ≤ 1). The mean TBR of the contralateral breast was 6.7 in the [^18^F]FDG PET/MRI group, ranging from 0.5 to 25.3. 6.9% (2/29) showed a TBR of the contralateral breast less than or equal to 1 (Fig. 3b).

#### Threshold based on the tumour-to-background ratio of the liver

For [^18^F]FDG PET/CT, the mean TBR of the liver was 2.1, ranging from 0.2 to 8.1. In 28.6% (34/119), the SUV_max_ of the primary tumour was lower than or equal to the SUV_max_ of the liver (TBR ≤ 1). The mean TBR of the liver was 1.7 in the [^18^F]FDG PET/MRI group, ranging from 0.2 to 4.7. In 35.5% (11/31), the TBR of the liver was less than or equal to 1 (Fig. 3b).

### Clinical factors that influence [^18^F] fluorodeoxyglucose uptake

Results of univariable and multivariable logistic regression for all metabolic thresholds of [^18^F]FDG PET/CT and [^18^F]FDG PET/MRI are provided in Table 3 and Supplementary File S2, Supplemental digital content 2, https://links.lww.com/NMC/A398, respectively. Concerning multivariable logistic regression of [^18^F]FDG PET/CT, the SUV_max_ of the primary breast tumour was more likely to be below 2.0 in case of a lobular histology (ILC vs. NST, adjusted OR: 4.93, 95% CI: 1.18–20.59). The SUV_max_ of the primary breast tumour was more likely to be below 2.5 in smaller breast tumours (cT2-4 vs. cT1, adjusted OR: 0.13, 95% CI: 0.03–0.53). Lobular histology, a smaller tumour, and a lower tumour grade were associated with SUV_max_ threshold 3.0 (ILC vs. NST, adjusted OR: 4.10, 95% CI: 1.26–13.33; cT2-2 vs. cT1, adjusted OR: 0.17, 95% CI: 0.05–0.65; grade 3 vs. 1–2, adjusted OR: 0.16, 95% CI: 0.03–0.80) as well as with the TBR of the liver (ILC vs. NST, adjusted OR: 5.72, 95% CI: 1.67–19.56; cT2-2 vs. cT1, adjusted OR: 0.19, 95% CI: 0.05–0.72; grade 3 vs. 1–2, adjusted OR: 0.20, 95% CI: 0.05–0.77). Regarding the TBR of the contralateral breast and the [^18^F]FDG PET/MRI group, the number of patients below each threshold was too low to perform multivariable regression.

**Table 3:** Univariable and multivariable logistic regression of variables associated with reduced [^18^F] fluorodeoxyglucose uptake, according to multiple baseline metabolic thresholds of the primary tumour on [^18^F] fluorodeoxyglucose PET/computed tomography

Threshold	Variables	Univariable logistic regression		Multivariable logistic regression	
		OR (95% CI)	*P* value	OR (95% CI)	*P* value
SUV_max_ 2.0	cT-status *(cT2-4 vs. cT1*)	0.29 (0.07–1.27)	0.100	0.26 (0.05–1.21)	0.085
	Number hypermetabolic ALNs	1.09 (0.86–1.38)	0.462		
	cN-status (*cN+ vs. cN0*)	0.59 (0.11–3.06)	0.530		
	PR status *(pos. vs. neg.*)	0.80 (0.21–3.02)	0.745		
	HER2 status *(pos. vs. neg.*)	0.00 (0.00 –.)	0.998		
	Histology type *(ILC vs. NST*)	4.48 (1.12–17.87)	**0.034**	4.93 (1.18–20.59)	**0.029**
	Tumour grade *(3 vs. 1–2*)	0.22 (0.03–1.78)	0.154		
	LVI *(yes vs. no*)	0.00 (0.00 –.)	0.999		
	Multifocal tumour *(yes vs. no*)	0.91 (0.24–3.42)	0.893		
SUV_max_ 2.5	cT-status *(cT2-4 vs. cT1*)	0.20 (0.06–0.65)	**0.007**	0.13 (0.03–0.53)	**0.004**
	Number hypermetabolic ALNs	1.03 (0.84–1.26)	0.785		
	cN-status (*cN+ vs. cN0*)	0.32 (0.10–1.06)	0.063		
	PR status *(pos. vs. neg.*)	0.83 (0.30–2.34)	0.729		
	HER2 status *(pos. vs. neg.*)	0.13 (0.02–1.05)	0.055		
	Histology type *(ILC vs. NST*)	3.67 (1.16–11.59)	**0.027**	3.14 (0.86–11.50)	0.084
	Tumour grade *(3 vs. 1–2*)	0.10 (0.01–0.74)	**0.025**	0.12 (0.14–1.04)	0.054
	LVI *(yes vs. no*)	0.00 (0.00 –.)	0.999		
	Multifocal tumour *(yes vs. no*)	0.65 (0.23–1.86)	0.420		
SUV_max_ 3.0	cT-status *(cT2-4 vs. cT1*)	0.26 (0.08–0.79)	**0.018**	0.17 (0.05–0.65)	**0.009**
	Number hypermetabolic ALNs	0.99 (0.83–1.19)	0.909		
	cN-status (*cN+ vs. cN0*)	0.29 (0.10–0.88)	**0.029**		
	PR status *(pos. vs. neg.*)	1.64 (0.63–4.29)	0.315		
	HER2 status *(pos. vs. neg.*)	0.29 (0.08–1.04)	0.058		
	Histology type *(ILC vs. NST*)	4.88 (1.69–14.11)	**0.003**	4.10 (1.26–13.33)	**0.019**
	Tumour grade *(3 vs. 1–2*)	0.12 (0.03–0.54)	**0.006**	0.16 (0.03–0.80)	**0.026**
	LVI *(yes vs. no*)	0.80 (0.15–4.14)	0.790		
	Multifocal tumour *(yes vs. no*)	0.43 (0.16–1.12)	0.083		
TBR of the contralateral breast ≤1	cT-status *(cT2-4 vs. cT1*)	0.12 (0.02–0.66)	**0.015**	Not feasible	
	Number hypermetabolic ALNs	1.17 (0.90–1.52)	0.252		
	cN-status (*cN+ vs. cN0*)	0.77 (0.08–7.08)	0.820		
	PR status *(pos. vs. neg.*)	1.11 (0.20–6.34)	0.906		
	HER2 status *(pos. vs. neg.*)	0.00 (0.00 –.)	0.998		
	Histology type *(ILC vs. NST*)	6.40 (1.18–34.70)	**0.031**		
	Tumour grade *(3 vs. 1–2*)	0.00 (0.00 –.)	0.998		
	LVI *(yes vs. no*)	0.00 (0.00 –.)	0.999		
	Multifocal tumour *(yes vs. no*)	0.67 (0.12–3.79)	0.648		
TBR of the liver parenchyma ≤1	cT-status *(cT2-4 vs. cT1*)	0.29 (0.10–0.88)	**0.029**	0.19 (0.05–0.72)	**0.015**
	Number hypermetabolic ALNs	0.97 (0.82–1.15)	0.732		
	cN-status (*cN+ vs. cN0*)	0.34 (0.12–0.99)	**0.048**		
	PR status *(pos. vs. neg.*)	1.76 (0.73–4.24)	0.206		
	HER2 status *(pos. vs. neg.*)	0.27 (0.09–0.85)	**0.025**	0.46 (0.12–1.70)	0.244
	Histology type *(ILC vs. NST*)	7.09 (2.39–21.05)	**<0.001**	5.72 (1.67–19.56)	**0.005**
	Tumour grade *(3 vs. 1–2*)	0.12 (0.03–0.43)	**0.001**	0.20 (0.05–0.77)	**0.020**
	LVI *(yes vs. no*)	1.50 (0.40–5.70)	0.552		
	Multifocal tumour *(yes vs. no*)	0.47 (0.20–1.10)	0.081		

Bold values indicate statistical significance (p < 0.05).

ALN, axillary lymph node; CI, confidence interval; cN-status, clinically nodal status; cT-status, clinically tumour status; HER2, human epidermal growth factor receptor 2; ILC, invasive lobular carcinoma; LVI, lymphovascular invasion; NST, no special type; OR, odds ratio; PR, progesterone receptor; SUV, standardised uptake value; TBR, tumour-to-background ratio.

### Survival analysis

In the [^18^F]FDG PET/CT group, median follow-up time for RFS was 61 months (range: 7–135 months). During follow-up, a recurrence was detected in 26.1% (31/119). The 5-year cumulative probability of RFS was 77.4% (95% CI: 69.8–85.9). In the [^18^F]FDG PET/MRI group, median follow-up time was 32 months (range: 6–111 months). A total of 12.9% (4/31) had a recurrence during follow-up. The 5-year cumulative probability of RFS was 72.5% (95% CI: 50.4–100.0).

**Fig. 4:**
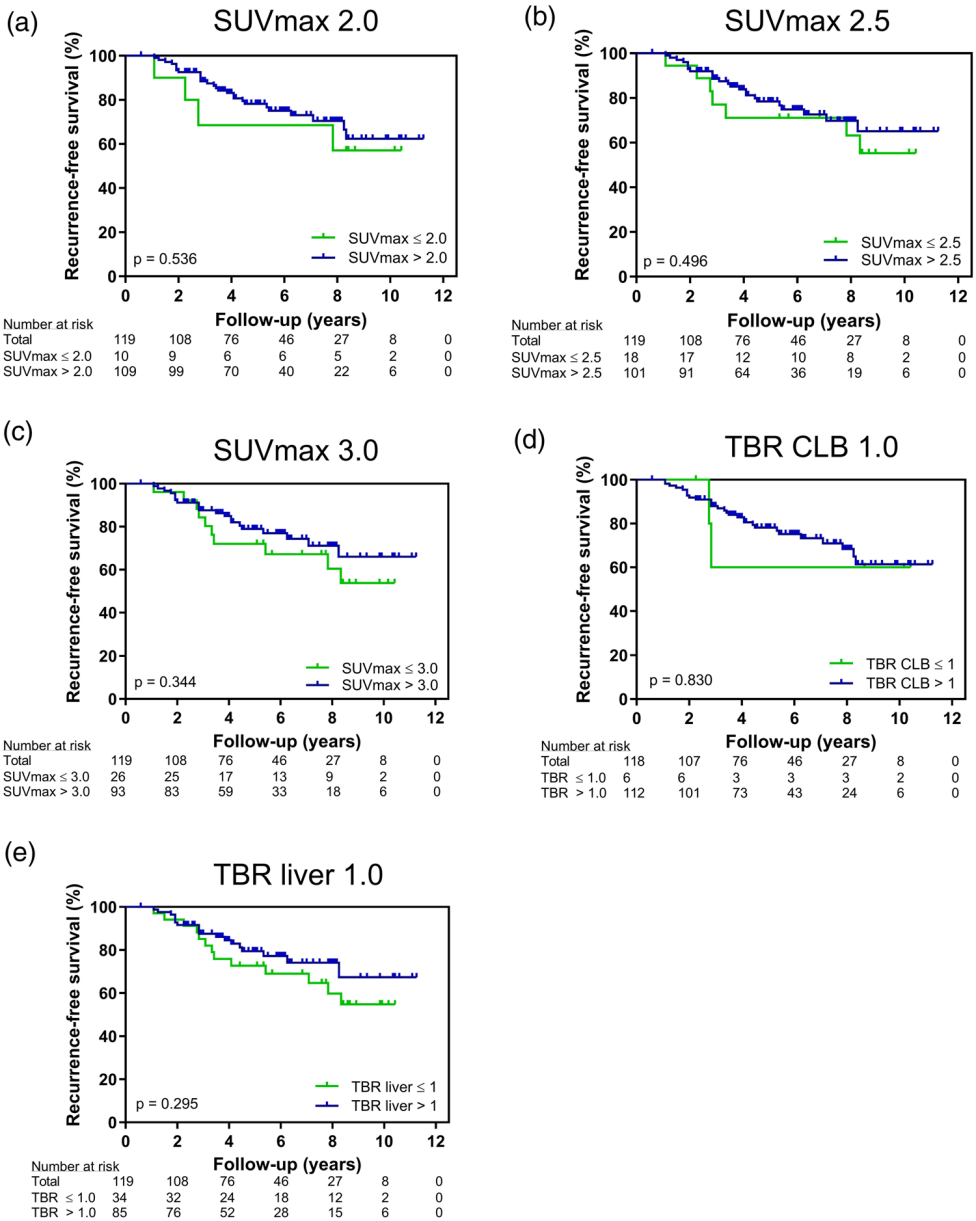
Kaplan–Meier plots of recurrence-free survival on [^18^F]FDG PET/CT for SUV_max_ threshold 2.0 (a), SUV_max_ threshold 2.5 (b), SUV_max_ threshold 3.0 (c), TBR of contralateral breast threshold 1.0 (d), and TBR of the liver threshold 1.0 (e). CT, computed tomography; [^18^F]FDG, [^18^F] fluorodeoxyglucose; SUV_max_, maximum standardised uptake value; TBR, tumour-to-background ratio.

The [^18^F]FDG PET/CT results for univariable Cox regression models applied to the clinical variables and PET-derived variables for RFS are presented in Table 4. Concerning RFS, age at diagnosis, axillary surgery type, pathologic complete response after NAC, and number of positive ALNs at surgery were significant predictors of recurrence. No PET-derived variables were predictors of RFS. Figure 4 shows no significant difference in RFS between patients with a metabolic activity below the threshold and above the threshold, for all applied metabolic thresholds. For the [^18^F]FDG PET/MRI group, only the Kaplan–Meier analysis was performed (Supplementary File S3, Supplemental digital content 3, https://links.lww.com/NMC/A399).

**Table 4:** Univariable Cox regression for recurrence-free survival of patients with [^18^F] fluorodeoxyglucose PET/computed tomography

Variables	Recurrence-free survival	
	HR (95% CI)	*P* value
Clinical variables		
Age at diagnosis	1.03 (1.00–1.06)	**0.035**
cT-status *(cT2-4 vs. cT1*)	1.07 (0.37–3.05)	0.905
cN-status (*cN+ vs. cN0*)	1.31 (0.40–4.30)	0.662
PR status *(pos. vs. neg.*)	0.50 (0.25–1.00)^a^	0.051
HER2 status *(pos. vs. neg.*)	0.89 (0.40–2.01)	0.782
Histology type *(ILC vs. NST*)	0.85 (0.30–2.43)	0.763
Tumour grade *(3 vs. 1–2*)	0.83 (0.36–1.91)	0.665
LVI *(yes vs. no*)	1.84 (0.65–5.24)^a^	0.251
Multifocal tumour *(yes vs. no*)	1.47 (0.73–2.98)	0.282
Therapy (*prim. surgery vs. NAC*)	1.66 (0.72–3.87)	0.238
Breast surgery *(mast. vs. lump.*)	1.29 (0.55–3.01)	0.556
Axillary surgery type		
None	Reference	
SLNB	0.07 (0.01–0.35)	**0.001**
SNLB/MARI	0.05 (0.01–0.44)	**0.008**
ALND	0.12 (0.04–0.44)	**0.001**
pCR after NAC (*yes vs. no*)	0.23 (0.05–0.97)	**0.046**
Radical surgery (*yes vs. no*)	1.00 (0.24–4.22)	0.998
Number N+ surgery	1.10 (1.03–1.16)	**0.002**
PET-derived variables		
SUV_max_ (*≤2.0 vs. >2.0*)	1.40 (0.48–4.06)	0.538
SUV_max_ (*≤2.5 vs. >2.5*)	1.34 (0.57–3.15)	0.499
SUV_max_ (*≤3.0 vs. >3.0*)	1.44 (0.67–3.07)	0.348
TBR contralat. breast (*≤1.0 vs. >1.*0)	1.17 (0.28–4.95)	0.831
TBR liver (*≤1.0 vs. >1.*0)	1.47 (0.71–3.02)	0.299
NT ratio	1.13 (0.82–1.55)	0.472
No. hypermetabolic ALN	1.04 (0.93–1.18)	0.490

Bold values indicate statistical significance (p < 0.05).

a Proportional hazards assumption violated.

ALN, axillary lymph node; ALND, axillary lymph node dissection; CI, confidence interval; cN-status, clinically nodal status; cT-status, clinically tumour status; HER2, human epidermal growth factor receptor 2; HR, hazard ratio; ILC, invasive lobular carcinoma; LVI, lymphovascular invasion; MARI, marking axillary lymph node with radioactive iodine seed; NAC, neoadjuvant chemotherapy; NST, no special type; NT ratio, nodal-to-tumour ratio; pCR, pathologic complete response; PR, progesterone receptor; SLNB, sentinel lymph node biopsy; SUV, standardised uptake value; TBR, tumour-to-background ratio.

## Discussion

Distant staging using ^18^F-FDG PET is often considered at the time of diagnosis in LABC, metastatic, or recurrent breast cancer. Previous studies have shown that estrogen receptor–positive breast tumours are not always detectable on PET, because of a low [^18^F]FDG uptake. This study aimed to investigate the proportion of women diagnosed with estrogen receptor–positive breast cancer who had a hypometabolic [^18^F]FDG uptake in the primary tumour on PET/CT or PET/MRI. Our analysis demonstrated that for every metabolic threshold, a considerable proportion of tumours fell below the applied thresholds. The proportion of hypometabolic tumours was lowest when the TBR of the contralateral breast was less than or equal to 1, and highest when the TBR of the liver parenchyma was less than or equal to 1. In addition, clinical factors influencing the metabolic activity toward a hypometabolic PET exam were identified for every metabolic threshold. In our study, a lower cT-status (cT1 vs. cT2-4), lobular histology type, and a lower tumour grade (1–2 vs. 3) were significantly correlated with a hypometabolic tumour for most metabolic thresholds. Lastly, the influence of a low [^18^F]FDG uptake on prognosis was assessed in terms of RFS. Survival analysis revealed no difference between patients above and below these metabolic thresholds, for all applied thresholds. However, given the propensity for late recurrences in low-grade, lobular tumours, the follow-up time in this cohort may have been too short.

To the best of our knowledge, this is the first study assessing the proportion of hypometabolic [^18^F]FDG uptake in estrogen receptor–positive breast cancer. Concerning clinical factors influencing the metabolic activity toward a hypometabolic PET exam, previous studies also found a positive correlation between SUV and tumour size, histological type (lower SUV_max_ in lobular breast cancer), and tumour grade [17,27,28]. Previous research and this study have demonstrated that tumours with a lower metabolic uptake are associated with clinical factors such as a lower tumour size, lower tumour grade, and no clinically suspected ALNs, which are indicative of a less aggressive form of breast cancer and, consequently, higher survival rates [29,30]. This may explain why potentially missing a metastasis from a hypometabolic tumour does not result in a significantly worse prognosis for these patients.

This study assessed multiple metabolic thresholds to attempt to determine the most suitable threshold for defining a hypometabolic tumour. Because this study has shown that a low metabolic activity does not appear to affect prognosis in this cohort, the optimal metabolic threshold for defining a hypometabolic tumour for clinical practice could not be determined. However, Groheux *et al*. [31] have stated that the SUV_max_ of the liver parenchyma is dependent on many factors such as patient age, weight, and blood glucose level. Therefore, TBR of the liver parenchyma may not be the most optimal metabolic threshold to select. Based on this data, TBR of the contralateral breast less than or equal to 1 could be a reasonable threshold; however, additional data are needed to confirm this, as only a small number of patients fell below the threshold in this group. Future studies are necessary to achieve an international consensus on the true definition of when a tumour is hypometabolic according to [^18^F]FDG PET.

The use of non-[^18^F]FDG PET tracers for patients with an estrogen receptor–positive tumour with a low metabolic activity is recommended according to the current National Comprehensive Cancer Network guideline [32]. [^18^F]fluoro-oestradiol ([^18^F]FES) is an estrogen-specific PET-tracer being effective for staging the recurrence of a previous estrogen receptor–positive breast cancer to verify whether estrogen receptor is still expressed in metastases and to assess the response to endocrine therapy [33]. However, as receptor expression can change over time, repeated biopsies are advised when using [^18^F]FES PET for response assessment [34]. In addition, Gallium 68-labelled fibroblast-activation protein inhibitor ([^68^Ga]FAPI) has recently emerged as a clinical PET tracer. [^68^Ga]FAPI binds to fibroblast activation protein (FAP), secreted by cancer-associated fibroblasts [35]. FAP is highly expressed in more than 90% of epithelial-derived tumours and their metastases, whereas not in healthy tissue, benign lesions, and precancerous tissues [36,37]. Multiple studies comparing [^68^Ga]FAPI with [^18^F]FDG in breast cancer reported a higher detection rate, higher SUV values, and higher TBRs with the former [38–40]. Other novel PET-tracers currently investigated for estrogen receptor–positive breast cancer include gastrin-releasing peptide receptor antagonists such as [^68^Ga]Ga-RM2, which has demonstrated utility particularly in low-grade breast cancer. In addition, [^18^F]fluoromisonidazole, which targets tumour hypoxia, is being explored [41,42]. Based on this information, several promising alternatives to [^18^F]FDG PET are emerging for staging of estrogen receptor–positive breast cancer patients.

This study has some limitations. First, the retrospective, single-centre study design may limit the generalizability of the findings. Second, no gene mutation analyses were performed, and histopathologic heterogeneity among tumours could have added further variability to the results. Third, as [^18^F]FDG PET/CT and [^18^F]FDG PET/MRI are different hybrid imaging modalities, the SUV_max_ values of both groups may not be comparable. Therefore, it was not possible to combine the two groups for analysis. The subgroup of [^18^F]FDG PET/MRI only consisted of 31 patients, which was too small to perform logistic regression and Cox regression. Fourth, volumetric PET parameters, such as metabolic tumour volume and total lesion glycolysis, were not assessed, which could have provided additional information. Fifth, the administered dose of [^18^F]FDG has changed during the study period from 2 to 3 MBq/kg. Lastly, concerning the survival analysis, the number of events may be too low, and the length of follow-up may be too short to detect metastases in low-grade, lobular tumours.

### Conclusion

This retrospective single-centre study found that a considerable proportion of estrogen receptor–positive LABC, metastatic, and recurrent breast cancer patients may have a hypometabolic tumour on [^18^F]FDG PET, although, the percentage depends on which metabolic threshold is applied. In patients with a lower cT-status, lobular histology and low-grade estrogen receptor–positive tumour, [^18^F]FDG PET may be less reliable as a staging procedure. Further research is necessary to determine the most appropriate metabolic threshold for defining a hypometabolic tumour on PET.

## Acknowledgements

Data were presented previously at the 14^th^ European Breast Cancer Conference and published as an abstract in the *European Journal of Cancer*.

### Conflicts of interest

T.J.A.v.N. received speaker honoraria, participation in medical advisory board meetings and institutional grant support from Bayer and GE Healthcare, and received consultancy agreement from Screenpoint Medical, not related to the content of this study. For the remaining authors, there are no conflicts of interest.

M.L.S. received institutional research funding not related to this study from Servier, Pharma, Nutricia and Illumina for the microbiota study. F.M.M. is supported by the German Research Foundation within the framework of Research Training Group 2375 (Tumour-Targeted Drug Delivery; Grant 331065168) and Clinical Research Unit 5011 (‘Integrating Emerging Methods to Advance Translational Kidney Research [InteraKD]’; Project 445703531) and reports institutional grants/contracts from GE Precision Healthcare LLC, NanoMab Technology Ltd., Radiopharm Ltd., and Siemens Healthcare not related to the study; and personal/consulting fees from Advanced Accelerator Applications (AAA) GmbH/Novartis, CURIUMTM, NanoMab Technology Ltd., and Telix Pharmaceuticals outside the submitted work. VCGTH reports institutional grants and personal fees from Roche, Novartis, Pfizer, Eli Lilly, and institutional grants from AstraZeneca, Daiichi Sankyo, and Gilead. F.A.G. is funded by Cancer Research UK, NVision, and AstraZeneca and has received research support and conference travel from GE Healthcare. F.A.G. and L.A. are supported by the NIHR Cambridge Biomedical Research Centre (NIHR203312). The views expressed are those of the authors and not necessarily those of the NIHR or the Department of Health and Social Care.

## Supplementary Material






