# Supplementary material for: The proportion, clinical predictors, and prognostic impact of hypometabolic estrogen receptor–positive primary breast cancer on baseline [18F] fluorodeoxyglucose PET
Source: Nucl Med Commun. 2026 Feb 25;47(6):704–14. doi: 10.1097/MNM.0000000000002133 (PMC13134659; doi:10.1097/MNM.0000000000002133)
Supplement: Supplementary file 1 [file nmc-47-704-s001.docx]

**Supplemental File 1.** Treatment regimen.

If patients were treated with neoadjuvant chemotherapy (NAC), treatment generally consisted of four cycles of 3-weekly doxorubicin and cyclophosphamide, followed by four cycles of 3-weekly docetaxel in case of an ER-positive and/or HER2 overexpressed tumor, or four cycles of weekly paclitaxel in case of a triple negative tumor. Patients with HER2 overexpressed tumors also received trastuzumab with/without pertuzumab. Surgery consisted of breast-conserving surgery (BCS) or mastectomy combined with surgery of the ipsilateral axilla (sentinel lymph node biopsy (SLNB) in case of clinically node negative (cN0), and axillary lymph node dissection (ALND) or SLNB combined with marking axillary lymph node with radioactive iodine seed (MARI) in case of clinical node positive (cN+) breast cancer. Pathologic complete response (pCR) of the primary tumor was defined as the absence of invasive cancer (ypT0/ypTis). Axillary pCR was defined as the absence of micrometastases (>0.2 mm and ≤2.0 mm) and macrometastases (>2.00 mm). Postoperative radiotherapy of the breast was performed after BCS. Chest wall and periclavicular irradiation were performed when patients had a cN1 (≥4 suspicious nodes), cT1-2ypN+, c/ypT3N+, c/ypT4, or c/ypN2–3 status. Solitary chest wall irradiation was performed in case of irradical breast surgery. The internal mammary chain (IMC) was irradiated in case of a PET-positive or a pathologically proven tumor-positive lymph node in IMC.
