# Supplementary material for: The proportion, clinical predictors, and prognostic impact of hypometabolic estrogen receptor–positive primary breast cancer on baseline [18F] fluorodeoxyglucose PET
Source: Nucl Med Commun. 2026 Feb 25;47(6):704–14. doi: 10.1097/MNM.0000000000002133 (PMC13134659; doi:10.1097/MNM.0000000000002133)
Supplement: Supplementary file 2 [file nmc-47-704-s002.docx]

**Supplemental File 2.**

**Table S1.** Univariable and multivariable logistic regression of variables associated with reduced [^18^F]FDG uptake, according to multiple baseline metabolic thresholds of the primary tumor on [^18^F]FDG PET/MRI.

|  |  | **Univariable Regression** | | **Multivariable Regression** | |
| --- | --- | --- | --- | --- | --- |
| **Threshold** | **Variables** | **OR (95% CI)** | **P-value** | **OR (95% CI)** | **P-value** |
| SUVmax 2.0 | cT status *(cT2-4 vs cT1)* | 0.01 (0.00 – 0.19) | **0.002** | Not feasible | |
|  | Number hypermetabolic ALNs | 0.32 (0.10 – 1.08) | 0.067 |  |  |
|  | cN status (*cN+ vs cN0)* | 0.20 (0.02 – 1.69) | 0.138 |  |  |
|  | PR status *(pos. vs neg.)* | 475139682 (0.00 – .) | 0.999 |  |  |
|  | HER2 status *(pos. vs neg.)* | 0.00 (0.00 – .) | 0.999 |  |  |
|  | Histology type *(ILC vs NST)* | 0.00 (0.00 – .) | 0.999 |  |  |
|  | Tumor grade *(3 vs 1-2)* | 0.00 (0.00 – .) | 0.999 |  |  |
|  | LVI *(yes vs no)* | 0.00 (0.00 – .) | 0.999 |  |  |
|  | Multifocal tumor *(yes vs no)* | 0.00 (0.00 – .) | 0.999 |  |  |
| SUVmax 2.5 | cT status *(cT2-4 vs cT1)* | 0.02 (0.00 – 0.29) | **0.004** | Not feasible | |
|  | Number hypermetabolic ALNs | 0.34 (0.12 – 1.00) | **0.049** |  |  |
|  | cN status (*cN+ vs cN0)* | 0.27 (0.03 – 2.19) | 0.221 |  |  |
|  | PR status *(pos. vs neg.)* | 2.35 (0.24 – 23.60) | 0.467 |  |  |
|  | HER2 status *(pos. vs neg.)* | 0.00 (0.00 – .) | 0.999 |  |  |
|  | Histology type *(ILC vs NST)* | 0.00 (0.00 – .) | 0.999 |  |  |
|  | Tumor grade *(3 vs 1-2)* | 0.00 (0.00 – .) | 0.999 |  |  |
|  | LVI *(yes vs no)* | 0.00 (0.00 – .) | 0.999 |  |  |
|  | Multifocal tumor *(yes vs no)* | 0.00 (0.00 – .) | 0.999 |  |  |
| SUVmax 3.0 | cT status *(cT2-4 vs cT1)* | 0.06 (0.01 – 0.66) | **0.021** | Not feasible | |
|  | Number hypermetabolic ALNs | 0.63 (0.37 – 1.09) | 0.098 |  |  |
|  | cN status (*cN+ vs cN0)* | 0.55 (0.08 – 4.04) | 0.559 |  |  |
|  | PR status *(pos. vs neg.)* | 1.63 (0.27 – 9.98) | 0.595 |  |  |
|  | HER2 status *(pos. vs neg.)* | 0.20 (0.02 – 1.94) | 0.166 |  |  |
|  | Histology type *(ILC vs NST)* | 2.25 (0.13 – 40.66) | 0.583 |  |  |
|  | Tumor grade *(3 vs 1-2)* | 0.20 (0.02 – 1.94) | 0.166 |  |  |
|  | LVI *(yes vs no)* | 0.00 (0.00 – .) | 0.999 |  |  |
|  | Multifocal tumor *(yes vs no)* | 0.29 (0.05 – 1.69) | 0.168 |  |  |
| TBR of the contralateral breast ≤1 | cT status *(cT2-4 vs cT1)* | 0.00 (0.00 – .) | 0.998 | Not feasible | |
|  | Number hypermetabolic ALNs | 0.29 (0.04 – 2.15) | 0.228 |  |  |
|  | cN status (*cN+ vs cN0)* | 140476067,64 (0.00 – .) | 0.999 |  |  |
|  | PR status *(pos. vs neg.)* | 179497199,61 (0.00 – .) | 0.999 |  |  |
|  | HER2 status *(pos. vs neg.)* | 0.00 (0.00 – .) | 0.999 |  |  |
|  | Histology type *(ILC vs NST)* | 0.00 (0.00 – .) | 1.000 |  |  |
|  | Tumor grade *(3 vs 1-2)* | 0.00 (0.00 – .) | 0.999 |  |  |
|  | LVI *(yes vs no)* | 0.00 (0.00 – .) | 0.999 |  |  |
|  | Multifocal tumor *(yes vs no)* | 0.00 (0.00 – .) | 0.999 |  |  |
| TBR of the liver parenchyma ≤1 | cT status *(cT2-4 vs cT1)* | 0.00 (0.00 – .) | 0.999 | Not feasible | |
|  | Number hypermetabolic ALNs | 0.73 (0.48 – 1.13) | 0.161 |  |  |
|  | cN status (*cN+ vs cN0)* | 0.79 (0.11 – 5.66) | 0.818 |  |  |
|  | PR status *(pos. vs neg.)* | 1.14 (0.22 – 5.87) | 0.873 |  |  |
|  | HER2 status *(pos. vs neg.)* | 0.38 (0.06 – 2.29) | 0.292 |  |  |
|  | Histology type *(ILC vs NST)* | 1.60 (0.09 – 28.57) | 0.749 |  |  |
|  | Tumor grade *(3 vs 1-2)* | 0.17 (0.02 – 1.58) | 0.119 |  |  |
|  | LVI *(yes vs no)* | 0.00 (0.00 – .) | 0.999 |  |  |
|  | Multifocal tumor *(yes vs no)* | 0.70 (0.15 – 3.17) | 0.642 |  |  |

OR, odds ratio; CI, confidence interval; SUV, standardized uptake value; TBR, tumor-to-background ratio; cT status, clinically tumor status; ALN, axillary lymph node; cN status, clinically nodal status; PR, progesterone receptor; HER2, human epidermal growth factor receptor 2; ILC, invasive lobular carcinoma; NST, no special type; LVI, lymphovascular invasion.

**
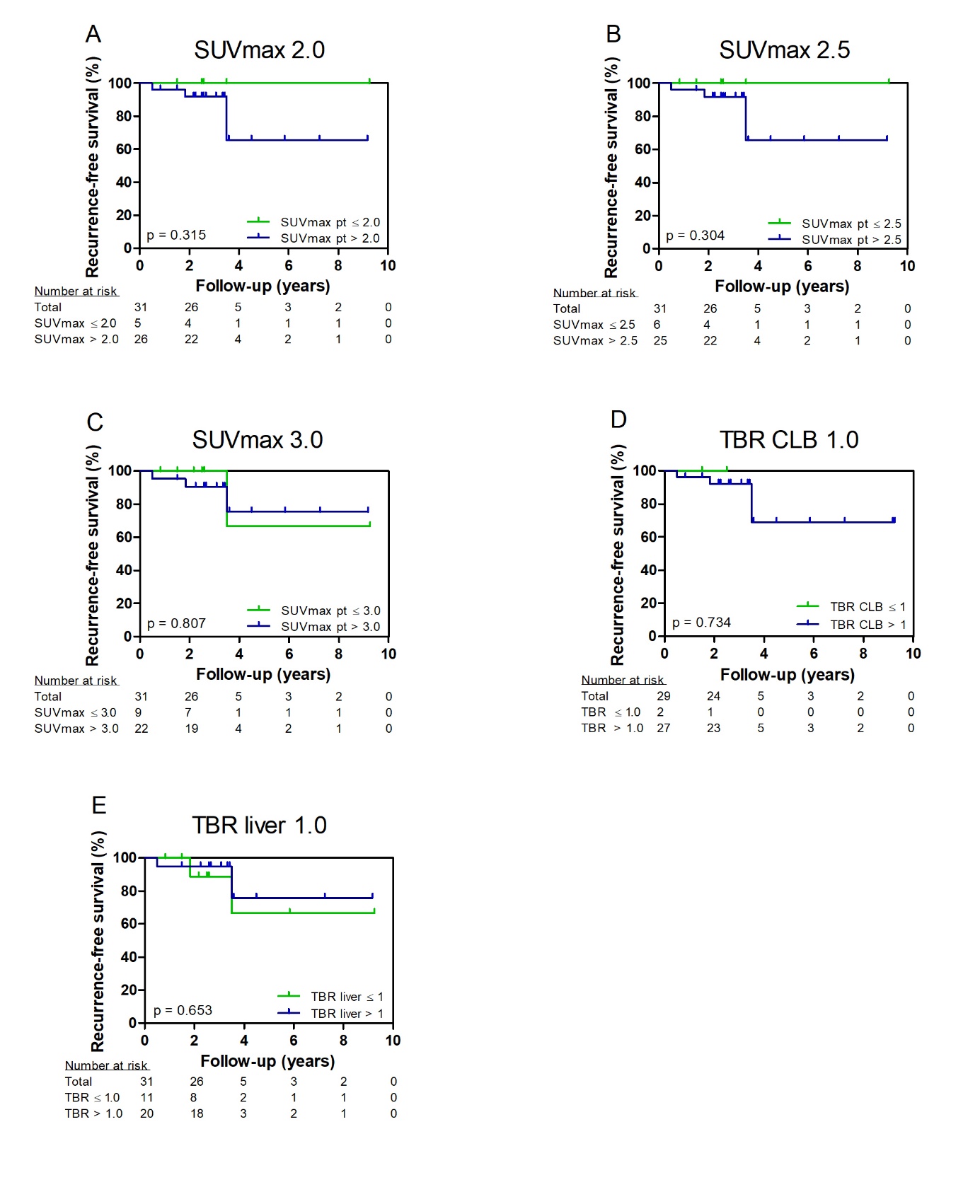
**

**Figure S1.** Kaplan Meier plots of RFS on [^18^F]FDG PET/MRI for SUVmax threshold 2.0 (A), SUVmax threshold 2.5 (B), SUVmax threshold 3.0 (C), TBR of contralateral breast threshold 1.0 (D), and TBR of the liver threshold 1.0 (E).
