# Supplementary material for: The proportion, clinical predictors, and prognostic impact of hypometabolic estrogen receptor–positive primary breast cancer on baseline [18F] fluorodeoxyglucose PET
Source: Nucl Med Commun. 2026 Feb 25;47(6):704–14. doi: 10.1097/MNM.0000000000002133 (PMC13134659; doi:10.1097/MNM.0000000000002133)
Supplement: Supplementary file 3 [file nmc-47-704-s003.docx]

**Appendix**

**Table 1.** SUVmax and SUVmean values of the primary tumor, contralateral breast, and liver parenchyma derived from [^18^F]FDG PET/CT.

| **Patient number** | **Primary tumor** | | **Contralateral breast** | | **Liver parenchyma** | |
| --- | --- | --- | --- | --- | --- | --- |
|  | **SUVmax** | **SUVmean** | **SUVmax** | **SUVmean** | **SUVmax** | **SUVmean** |
| 1 | 1,86 | 1,16 | 1,67 | 0,76 | 2,67 | 1,88 |
| 2 | 5,16 | 1,39 | 1,20 | 0,71 | 3,00 | 1,97 |
| 3 | 7,97 | 2,79 | 0,77 | 0,34 | 3,26 | 2,26 |
| 4 | 10,52 | 3,41 | 0,66 | 0,30 | 3,47 | 2,32 |
| 5 | 10,81 | 2,97 | 1,97 | 1,19 | 3,63 | 2,61 |
| 6 | 3,01 | 1,43 | 1,15 | 0,36 | 3,31 | 2,29 |
| 7 | 3,97 | 1,01 | 2,08 | 0,90 | 2,87 | 1,95 |
| 8 | 2,84 | 1,38 | 1,50 | 0,61 | 3,32 | 2,42 |
| 9 | 3,21 | 1,49 | 1,74 | 1,08 | 3,37 | 2,37 |
| 10 | 3,20 | 1,18 | 0,91 | 0,52 | 3,27 | 2,37 |
| 11 | 1,17 | 0,64 | 1,36 | 0,62 | 3,70 | 2,61 |
| 12 | 9,74 | 3,52 | 1,68 | 0,84 | 3,05 | 2,20 |
| 13 | 7,71 | 1,64 | 1,72 | 0,63 | 2,96 | 2,12 |
| 14 | 2,91 | 0,95 | 1,95 | 0,83 | 5,99 | 3,94 |
| 15 | 3,70 | 1,77 | 1,02 | 0,47 | 3,24 | 2,27 |
| 16 | 4,86 | 2,77 | 1,63 | 1,16 | 3,07 | 2,26 |
| 17 | 5,50 | 2,84 | 1,65 | 0,85 | 3,24 | 2,35 |
| 18 | 2,01 | 1,07 | 1,95 | 1,00 | 3,53 | 2,45 |
| 19 | 2,03 | 1,07 | 1,60 | 0,48 | 3,29 | 2,40 |
| 20 | 6,59 | 2,10 | 0,54 | 0,25 | 2,46 | 1,73 |
| 21 | 8,65 | 2,62 | 1,75 | 0,75 | 3,83 | 2,66 |
| 22 | 5,29 | 1,62 | 1,83 | 1,10 | 2,94 | 2,17 |
| 23 | 3,30 | 1,60 | 1,94 | 0,49 | 3,96 | 2,58 |
| 24 | 2,23 | 0,87 | 1,56 | 0,59 | 3,00 | 1,88 |
| 25 | 2,91 | 1,69 | 2,10 | 0,89 | 3,03 | 2,31 |
| 26 | 3,57 | 1,27 | 2,14 | 0,94 | 3,64 | 2,37 |
| 27 | 4,63 | 1,08 | 1,46 | 0,42 | 3,05 | 2,18 |
| 28 | 3,72 | 0,64 | 0,58 | 0,26 | 4,29 | 2,58 |
| 29 | 9,25 | 3,24 | 1,60 | 0,71 | 3,14 | 2,08 |
| 30 | 20,81 | 5,28 | 1,68 | 0,40 | 4,35 | 2,89 |
| 31 | 6,09 | 1,93 | 1,01 | 0,42 | 3,98 | 2,43 |
| 32 | 5,04 | 1,47 | 0,43 | 0,25 | 4,32 | 3,06 |
| 33 | 8,84 | 1,81 | 2,44 | 0,52 | 3,42 | 2,38 |
| 34 | 5,08 | 1,43 | 1,47 | 0,61 | 3,07 | 2,25 |
| 35 | 9,84 | 2,49 | 2,15 | 0,63 | 3,52 | 2,41 |
| 36 | 2,38 | 0,91 | 0,99 | 0,34 | 3,57 | 2,14 |
| 37 | 18,84 | 3,96 | 0,99 | 0,33 | 3,27 | 3,22 |
| 38 | 5,64 | 2,10 | 2,49 | 1,29 | 3,59 | 2,69 |
| 39 | 11,71 | 2,43 | 1,59 | 0,42 | 3,48 | 2,27 |
| 40 | 6,48 | 1,99 | 2,18 | 0,79 | 3,46 | 2,13 |
| 41 | 2,50 | 1,62 | 1,24 | 0,57 | 3,23 | 2,24 |
| 42 | 4,92 | 2,63 | 2,41 | 0,68 | 4,37 | 2,90 |
| 43 | 4,54 | 2,32 | 2,19 | 1,09 | 3,82 | 2,56 |
| 44 | 2,95 | 2,10 | 1,28 | 0,74 | 3,94 | 2,51 |
| 45 | 10,46 | 2,95 | 0,64 | 0,30 | 3,09 | 2,05 |
| 46 | 2,40 | 1,09 | 1,69 | 0,86 | 3,18 | 2,35 |
| 47 | 3,10 | 1,44 | 1,80 | 0,68 | 3,55 | 2,48 |
| 48 | 5,15 | 2,74 | 1,21 | 0,39 | 3,36 | 2,35 |
| 49 | 13,18 | 5,31 | 1,05 | 0,77 | 2,60 | 2,00 |
| 50 | 3,86 | 1,12 | 0,76 | 0,43 | 3,43 | 2,41 |
| 51 | 24,43 | 3,75 | 2,22 | 1,16 | 4,34 | 3,19 |
| 52 | 9,41 | 2,66 | 1,52 | 0,91 | 2,64 | 1,88 |
| 53 | 5,08 | 2,76 | 1,32 | 0,43 | 3,34 | 2,35 |
| 54 | 3,87 | 1,80 | 1,47 | 0,77 | 2,94 | 1,76 |
| 55 | 1,66 | 1,38 | 1,10 | 0,74 | 2,60 | 1,92 |
| 56 | 2,46 | 1,00 | 1,30 | 0,52 | 3,14 | 2,18 |
| 57 | 4,81 | 1,16 | 1,76 | 0,54 | 3,93 | 2,83 |
| 58 | 13,79 | 2,89 | 1,73 | 0,74 | 3,92 | 2,99 |
| 59 | 3,16 | 1,95 | 1,67 | 1,22 | 2,77 | 2,16 |
| 60 | 2,20 | 1,40 | 1,20 | 0,50 | 3,70 | 2,60 |
| 61 | 1,50 | 0,90 | 1,00 | 0,30 | 3,00 | 1,90 |
| 62 | 9,50 | 7,20 | 1,00 | 0,50 | 3,50 | 2,20 |
| 63 | 3,40 | 1,90 | 0,50 | 0,20 | 2,70 | 1,70 |
| 64 | 1,05 | 0,54 | 1,03 | 0,42 | 4,31 | 2,32 |
| 65 | 13,40 | 7,60 | 1,20 | 0,80 | 2,10 | 1,20 |
| 66 | 2,94 | 1,23 | 1,13 | 0,45 | 3,27 | 2,37 |
| 67 | 2,13 | 1,27 | 2,09 | 0,97 | 3,28 | 2,25 |
| 68 | 6,49 | 2,68 | 1,63 | 0,86 | 3,50 | 2,37 |
| 69 | 19,95 | 4,46 | 1,45 | 0,57 | 4,23 | 2,77 |
| 70 | 5,68 | 1,23 | 2,09 | 0,68 | 3,55 | 2,65 |
| 71 | 18,29 | 4,41 | 1,50 | 0,53 | 3,05 | 2,31 |
| 72 | 11,32 | 1,35 | 1,71 | 0,41 | 3,87 | 2,85 |
| 73 | 15,77 | 11,08 | 2,34 | 1,02 | 3,35 | 2,66 |
| 74 | 3,92 | 2,66 | 0,91 | 1,33 | 3,39 | 2,61 |
| 75 | 8,04 | 4,51 | 1,39 | 0,69 | 2,90 | 2,26 |
| 76 | 12,18 | 7,22 | 1,43 | 0,43 | 3,13 | 2,42 |
| 77 | 2,49 | 1,35 | 1,70 | 0,91 | 3,01 | 2,36 |
| 78 | 7,41 | 4,13 | 2,06 | 0,74 | 4,05 | 2,94 |
| 79 | 11,78 | 8,82 | 1,36 | 0,58 | 3,56 | 2,85 |
| 80 | 10,88 | 4,17 | 1,84 | 0,66 | 3,52 | 2,76 |
| 81 | 7,61 | 3,17 | 1,66 | 0,90 | 3,36 | 2,53 |
| 82 | 2,92 | 1,67 | 1,70 | 0,67 | 3,43 | 2,27 |
| 83 | 6,08 | 3,46 | 1,78 | 0,47 | 3,70 | 2,64 |
| 84 | 6,75 | 3,63 | 1,69 | 0,89 | 3,83 | 2,46 |
| 85 | 11,36 | 2,02 | 0,88 | 0,36 | 3,58 | 2,64 |
| 86 | 7,97 | 4,45 | 2,05 | 0,93 | 3,35 | 2,60 |
| 87 | 1,49 | 0,49 | 0,66 | 0,23 | 3,51 | 2,22 |
| 88 | 11,04 | 4,92 | 1,80 | 1,43 | 3,63 | 2,78 |
| 89 | 7,23 | 5,63 | 1,79 | 1,03 | 3,18 | 2,39 |
| 90 | 4,21 | 2,67 | 1,99 | 0,87 | 3,74 | 2,68 |
| 91 | 6,28 | 4,24 | 1,62 | 1,30 | 3,29 | 2,59 |
| 92 | 9,32 | 6,94 | 2,12 | 1,36 | 2,85 | 2,05 |
| 93 | 3,23 | 2,16 | 1,09 | 0,38 | 3,60 | 2,65 |
| 94 | 4,78 | 3,17 | 1,90 | 0,68 | 3,24 | 2,39 |
| 95 | 12,38 | 7,65 | 0,90 | 0,35 | 3,59 | 2,79 |
| 96 | 5,10 | 3,33 | 0,63 | 0,18 | 4,00 | 2,93 |
| 97 | 21,50 | 4,19 | 2,31 | 0,58 | 3,95 | 2,75 |
| 98 | 27,69 | 15,52 | 0,89 | 0,42 | 3,41 | 2,35 |
| 99 | 6,09 | 3,94 | 1,89 | 0,78 | 3,06 | 2,31 |
| 100 | 3,34 | 2,35 | 1,74 | 0,70 | 3,68 | 2,86 |
| 101 | 13,18 | 9,29 | 0,62 | 0,27 | 3,64 | 2,86 |
| 102 | 1,82 | 1,25 | 2,16 | 0,60 | 4,23 | 3,29 |
| 103 | 7,16 | 4,60 | 2,27 | 1,57 | 3,45 | 2,48 |
| 104 | 7,96 | 5,18 | 2,00 | 0,77 | 3,71 | 2,69 |
| 105 | 2,83 | 1,25 | 1,85 | 0,67 | 3,60 | 2,75 |
| 106 | 6,36 | 5,37 | 2,42 | 1,34 | 3,28 | 2,35 |
| 107 | 8,55 | 6,07 | 0,76 | 0,44 | 4,10 | 2,80 |
| 108 | 4,01 | 3,23 | 2,42 | 1,45 | 2,79 | 2,13 |
| 109 | 5,63 | 3,14 | 1,99 | 1,15 | 3,02 | 2,33 |
| 110 | 7,12 | 2,51 | 0,98 | 0,30 | 4,12 | 2,99 |
| 111 | 1,53 | 1,03 | 1,93 | 0,99 | 3,55 | 2,65 |
| 112 | 6,90 | 2,89 | 1,40 | 0,30 | 3,40 | 2,40 |
| 113 | 11,00 | 7,70 | 2,30 | 0,60 | 3,40 | 2,60 |
| 114 | 4,56 | 2,81 | **—** | **—** | 3,70 | 2,76 |
| 115 | 22,62 | 6,50 | 0,61 | 0,28 | 3,37 | 2,55 |
| 116 | 10,01 | 4,29 | 1,92 | 0,60 | 3,15 | 2,39 |
| 117 | 12,34 | 5,01 | 1,65 | 0,63 | 3,51 | 2,69 |
| 118 | 4,70 | 2,80 | 0,90 | 0,40 | 3,10 | 2,50 |
| 119 | 4,30 | 0,86 | 0,98 | 0,42 | 3,33 | 2,45 |

**—** Unable to measure

SUVmax, maximum standardized uptake value; SUVmean, mean standardized uptake value.

**Table 2.** SUVmax and SUVmean values of the primary tumor, contralateral breast, and liver parenchyma derived from [^18^F]FDG PET/MRI

| **Patient number** | **Primary tumor** | | **Contralateral breast** | | **Liver parenchyma** | |
| --- | --- | --- | --- | --- | --- | --- |
|  | **SUVmax** | **SUVmean** | **SUVmax** | **SUVmean** | **SUVmax** | **SUVmean** |
| 1 | 8,45 | 3,69 | 1,33 | 0,39 | 3,95 | 1,97 |
| 2 | 7,40 | 5,00 | 0,70 | 0,30 | 4,90 | 2,40 |
| 3 | 5,13 | 3,74 | 0,40 | 0,10 | 4,40 | 2,50 |
| 4 | 5,00 | 2,22 | 2,16 | 1,18 | 3,80 | 1,94 |
| 5 | 10,10 | 6,70 | 0,40 | 0,30 | 2,90 | 1,80 |
| 6 | 5,60 | 3,50 | 1,60 | 1,00 | 3,50 | 2,30 |
| 7 | 6,70 | 4,25 | 1,00 | 0,50 | 3,90 | 2,40 |
| 8 | 7,80 | 6,00 | 0,70 | 0,50 | 3,50 | 2,30 |
| 9 | 12,70 | 7,30 | 0,70 | 0,30 | 2,70 | 1,80 |
| 10 | 3,30 | 2,50 | 0,60 | 0,30 | 3,60 | 2,40 |
| 11 | 1,20 | 0,90 | 1,30 | 0,40 | 4,20 | 2,70 |
| 12 | 5,50 | 2,70 | 1,10 | 0,60 | 3,40 | 2,10 |
| 13 | 2,80 | 1,60 | **—** | **—** | 3,50 | 2,30 |
| 14 | 11,00 | 8,60 | 0,80 | 0,40 | 2,90 | 1,80 |
| 15 | 5,70 | 3,30 | 1,70 | 0,80 | 4,00 | 2,80 |
| 16 | 6,90 | 4,40 | 1,60 | 0,80 | 3,10 | 2,00 |
| 17 | 2,70 | 1,40 | 0,80 | 0,20 | 3,70 | 2,60 |
| 18 | 0,50 | 0,30 | 1,00 | 0,40 | 2,80 | 1,80 |
| 19 | 9,20 | 6,10 | 0,50 | 0,20 | 3,10 | 1,80 |
| 20 | 11,00 | 9,70 | 1,40 | 0,70 | 3,60 | 2,40 |
| 21 | 12,90 | 6,70 | 2,60 | 0,70 | 3,50 | 2,80 |
| 22 | 2,90 | 1,20 | 1,10 | 0,40 | 4,60 | 2,50 |
| 23 | 5,40 | 3,40 | 2,50 | 1,60 | 2,80 | 2,30 |
| 24 | 2,40 | 1,10 | 1,70 | 0,80 | 3,40 | 2,70 |
| 25 | 3,40 | 1,90 | 1,10 | 0,80 | 3,00 | 2,30 |
| 26 | 1,85 | 1,01 | 1,24 | 0,67 | 2,80 | 2,09 |
| 27 | 3,13 | 1,12 | 1,28 | 0,63 | 3,89 | 2,17 |
| 28 | 6,10 | 3,60 | 1,60 | 1,00 | 3,90 | 2,90 |
| 29 | 1,80 | 1,00 | **—** | **—** | 3,10 | 1,90 |
| 30 | 1,50 | 0,90 | 1,00 | 0,70 | 2,70 | 1,60 |
| 31 | 18,92 | 5,71 | 1,69 | 0,86 | 6,41 | 3,00 |

**—** Unable to measure

SUVmax, maximum standardized uptake value; SUVmean, mean standardized uptake value.
